# ApoE-associated modulation of neuroprotection from Aβ-mediated neurodegeneration in transgenic *Caenorhabditis elegans*

**DOI:** 10.1242/dmm.037218

**Published:** 2019-02-15

**Authors:** Edward F. Griffin, Samuel E. Scopel, Cayman A. Stephen, Adam C. Holzhauer, Madeline A. Vaji, Ryan A. Tuckey, Laura A. Berkowitz, Kim A. Caldwell, Guy A. Caldwell

**Affiliations:** 1 Department of Biological Sciences, The University of Alabama, Box 870344, Tuscaloosa, AL 35487-0344, USA; 2 Departments of Neurology and Neurobiology, Center for Neurodegeneration and Experimental Therapeutics, Nathan Shock Center for Research on the Basic Biology of Aging, University of Alabama at Birmingham School of Medicine, Birmingham, AL 35294, USA

**Keywords:** ApoE, Aβ, Neurodegeneration, Alzheimer's disease, *C*. *elegans*

## Abstract

Allele-specific distinctions in the human apolipoprotein E (*APOE*) locus represent the best-characterized genetic predictor of Alzheimer's disease (AD) risk. Expression of isoform *APOE*ε2 is associated with reduced risk, while *APOE*ε3 is neutral and *APOE*ε4 carriers exhibit increased susceptibility. Using *Caenorhabditis elegans*, we generated a novel suite of humanized transgenic nematodes to facilitate neuronal modeling of amyloid-beta peptide (Aβ) co-expression in the context of distinct human *APOE* alleles. We found that co-expression of human *APOE*ε2 with Aβ attenuated Aβ-induced neurodegeneration, whereas expression of the *APOE*ε4 allele had no effect on neurodegeneration, indicating a loss of neuroprotective capacity. Notably, the *APOE*ε3 allele displayed an intermediate phenotype; it was not neuroprotective in young adults but attenuated neurodegeneration in older animals. There was no functional impact from the three *APOE* isoforms in the absence of Aβ co-expression. Pharmacological treatment that examined neuroprotective effects of *APOE* alleles on calcium homeostasis showed allele-specific responses to changes in ER-associated calcium dynamics in the Aβ background. Additionally, Aβ suppressed survival, an effect that was rescued by *APOE*ε2 and *APOE*ε3, but not *APOE*ε4. Expression of the *APOE* alleles in neurons, independent of Aβ, exerted no impact on survival. Taken together, these results illustrate that *C. elegans* provides a powerful *in vivo* platform with which to explore how AD-associated neuronal pathways are modulated by distinct *APOE* gene products in the context of Aβ-associated neurotoxicity. The significance of both ApoE and Aβ to AD highlights the utility of this new pre-clinical model as a means to dissect their functional inter-relationship.

This article has an associated First Person interview with the first author of the paper.

## Abstract (precis)

**Summary:** We report new humanized nematode models of amyloid-beta and apolipoprotein E gene expression, examining readouts for neurodegeneration, behavior, cellular function and survival, to elucidate the relative contributions of the proteins to the underlying pathology of Alzheimer's disease.

## INTRODUCTION

Alzheimer's disease (AD), characterized by the formation of insoluble amyloid-beta peptide (Aβ) plaques in the brain, accounts for nearly 70% of all late-life dementia. Although the causes, whether genetic or environmental, are not clearly defined, it is evident that the most predictive genetic association is variation in the gene encoding apolipoprotein E (ApoE). Although estimates vary based on study and ethnicity, ∼40% of AD cases harbor the ε4 allele of *APOE* (Spinney, 2014). This allele is a significant risk factor for late-onset AD, where two copies of *APOE*ε4 increases AD risk up to 15-fold relative to *APOE*ε3. The *APOE*ε2 allele appears to provide protection against AD via a mechanism that consists of more than the absence of the *APOE*ε4 allele (Corder et al., 1994; Talbot et al., 1994). Indeed, there may be opposing actions of the *APOE*ε2 and *APOE*ε4 alleles, which would not be unprecedented, as *APOE*ε2 and *APOE*ε4 appear to have opposing activities in lipidation and aggregate stabilization (Hu et al., 2015). Despite this correlation, the mechanisms by which differences in *APOE* allelic function modify AD risk are not entirely understood.

There are many mechanisms proposed to explain how *APOE*ε4 increases AD risk, including altered glucose and lipid metabolism. Most commonly, however, Aβ-dependent effects are considered within the context of the *APOE* alleles, where neurotoxicity and aggregation are examined. For example, mammalian models have yielded significant information on how ApoE and Aβ interact to affect cellular function and animal behavior, but the scale and complexity of the mammalian nervous system frustrate examination of quantifiable effects on individual neurons and their functional connectivity. The nematode *Caenorhabditis elegans* has been employed to generate models of neurodegenerative disorders, including AD (Griffin et al., 2017), Huntington's disease (Muñoz-Lobato et al., 2014) and Parkinson's disease (Martinez et al., 2017a). Because *C. elegans* is the only animal for which a connectivity map of its entire nervous system exists, it provides an unparalleled platform for the examination and quantitative characterization of neural interactions. Further, the genetic tractability of *C. elegans* offers a model receptive to genetic manipulation and transgenics. Importantly, specific worm models have proven highly predictive of both genetic and small molecule modifier results obtained in mammalian systems, including genome-wide association studies and induced pluripotent stem cells from patients (Cooper et al., 2006; Matlack et al., 2014; Mazzulli et al., 2011; Su et al., 2010; Tardiff et al., 2013, 2017; Treusch et al., 2011).

Here, we present new neuronal models to assay ApoE activity *in vivo* that consist of nematodes expressing human *APOE*ε2, *APOE*ε3 or *APOE*ε4 along with Aβ. Glutamate is a major excitatory neurotransmitter in the brain, and dysregulation of the glutamatergic system can lead to excitotoxicity, which, when chronic, has been hypothesized to play a role in neurodegeneration (Lewerenz and Maher, 2015). Because the glutamatergic circuitry is severely disrupted in the brains of AD patients (Francis et al., 1993; Greenamyre et al., 1988), the *eat-4* (glutamate transporter) promoter was chosen for glutamatergic neuron-specific expression of Aβ and the respective *APOE* alleles. Effects on neuronal integrity were examined through quantitative fluorescent imaging of neurodegeneration and behavioral assays. Additionally, we modulated neurodegenerative effectors via pharmacological treatment and RNA interference (RNAi). By combining neuronal expression of *APOE* alleles with a transgenic nematode model of human Aβ toxicity, we can further understand the clinically significant relationship between ApoE and Aβ in neurotoxicity. Using these *C. elegans* models of progressive Aβ-mediated neurodegeneration, a strong attenuation of Aβ-mediated toxicity is revealed by the *APOE*ε2 allele, as well as a modest, yet significant, intermediate protection phenotype by *APOE*ε3 as animals age, *in vivo*. Strikingly, the neuroprotective activity of ApoE was abolished in animals co-expressing Aβ and *APOE*ε4. Furthermore, this shows that the allelic profile reflects the well-established clinical observation of ApoE-associated susceptibility. Pharmacological and post-transcriptional manipulation further demonstrate differential activities of *APOE* alleles observable through multiple phenotypic outputs. Though limited as an invertebrate system, *C. elegans* provides a platform that accelerates attainment of a more mechanistic understanding of how ApoE protein variants function to modulate neuronal degeneration and establishes a new pre-clinical model of AD to accelerate future drug discovery.

## RESULTS

### *APOE* allele-selective mitigation of Aβ-mediated neurodegeneration

The Aβ peptide is the product of sequential cleavage of the amyloid precursor protein (APP) either at the cell surface or within endosomes. Cleavage of APP is known to produce multiple peptide products, such as Aβ(1-40) and Aβ(1-42); however, the Aβ(1-42) peptide is the most toxic. Extracellular deposition of insoluble Aβ plaques is a pathological hallmark of AD, but intracellular Aβ has been shown to be far more toxic (Burdick et al., 1992; Cha et al., 2012; Esbjörner et al., 2014; Hu et al., 2009; Kounnas et al., 1995; Li et al., 2012; Liu et al., 2013b; Naj et al., 2011; Nakagawa et al., 2000; Okoshi et al., 2015; Reinders et al., 2016; Snyder et al., 2005; Takahashi et al., 2002; Treusch et al., 2011; Ulrich, 2015; Wang et al., 2000; Yang et al., 1998; Zhao et al., 2015). To reproduce the intracellular accumulation of Aβ in *C. elegans*, Aβ was cloned with promoters for tissue-specific multicopy expression and scored for toxicity. In *C. elegans* muscle expression models of Aβ toxicity, Aβ was found to form plaques (Link et al., 2001) and intramuscular inclusions (Fay et al., 1998; Link, 1995), and to induce paralysis via cytotoxicity (Dostal and Link, 2010; Fonte et al., 2002). Furthermore, we have shown that expression of Aβ in glutamatergic neurons results in progressive, age-dependent, neurodegeneration modulated by endocytic and endosomal regulators, including the established AD modifier PICALM (Griffin et al., 2018; Treusch et al., 2011), and is amenable to pharmacological treatment (Matlack et al., 2014; Tardiff et al., 2017). To examine the relationship between ApoE and Aβ, we utilized a *C. elegans* model in which an Aβ(1-42) construct, hereafter referred to as Aβ, was cloned for expression in the glutamatergic neurons and neurodegeneration was quantified with precision in the five glutamatergic neurons in the tail (Matlack et al., 2014; Treusch et al., 2011). Expression in the glutamatergic neurons was achieved using the promoter for the glutamate transporter *eat-4*, which does not significantly change in expression across larval stages (Lee et al., 1999).

**Fig. 1.**
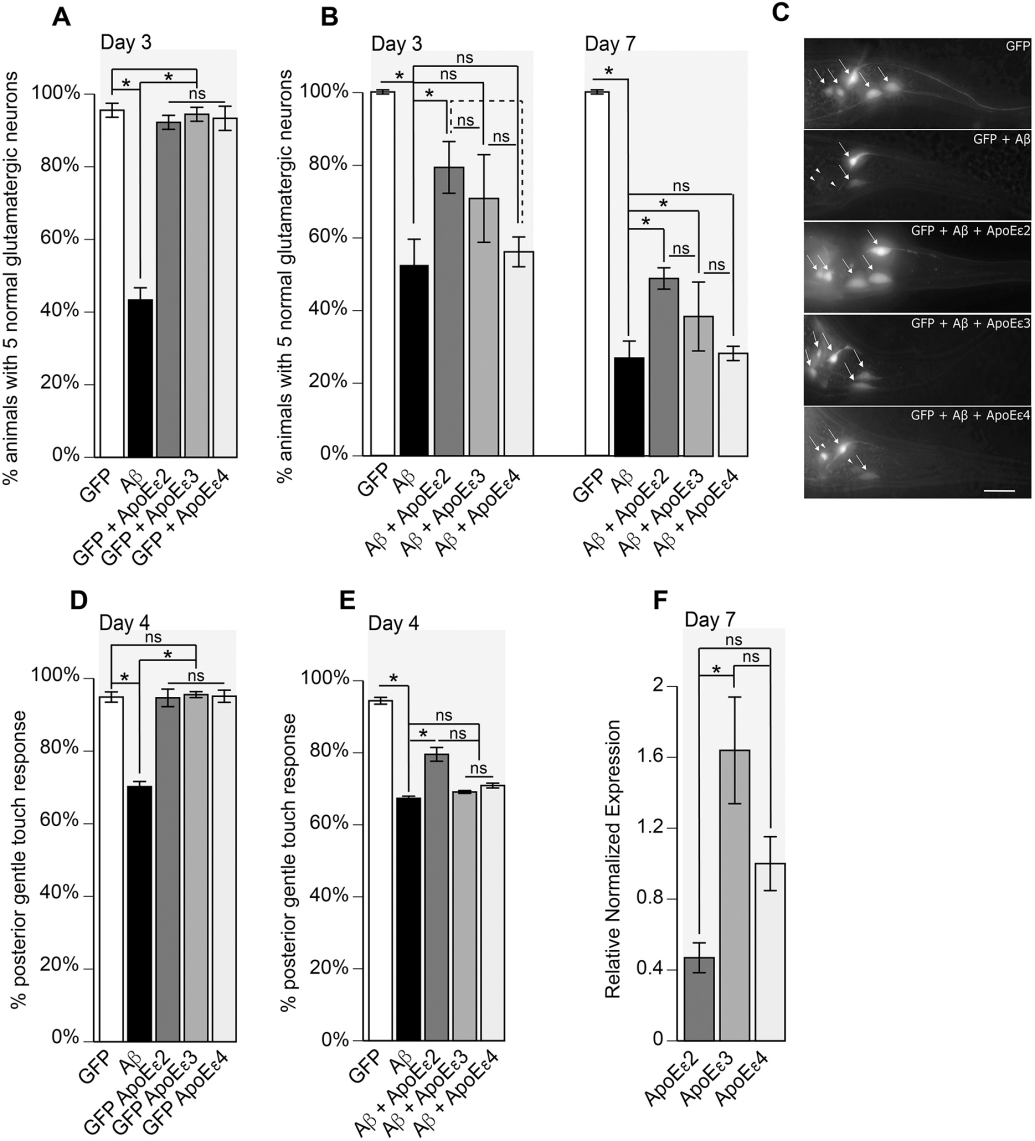
**Overexpression of Aβ induces neurodegeneration that is mitigated by ApoEε2 and ApoEε3, but not ApoEε4.** (A) Expression of GFP from the *eat-4* promoter {strain DA1240(*adIs1240*[P*_eat-4_*::GFP+*lin-15*(+)])} illuminates the glutamatergic neurons. The five tail glutamatergic neurons are assayed for neurodegeneration. Glutamatergic co-expression of Aβ {UA198(*baIn34*[P*_eat-4_*::Aβ,P*_myo-2_*::mCherry]; *adIs1240*[P*_eat-4_*::GFP])} induces neurodegeneration in synchronized hermaphrodite populations at day 3 post-hatching (*P*<0.0001), while overexpression of *APOE*ε2 {UA356 (*adIs1240*[P*_eat-4_*::GFP+*lin-15*(+)]; *baIn50*[P*_eat-4_*::*APOE*ε2, P*_unc-54_*::tdTomato])}, *APOE*ε3 {UA357 (*adIs1240*[P*_eat-4_*::GFP+*lin-15*(+)]; *baIn51*[P*_eat-4_*::*APOE*ε3, P*_unc-54_*::tdTomato])} or *APOE*ε4 {UA358 (*adIs1240*[P*_eat-4_*::GFP+*lin-15*(+)]; *baIn52*[P*_eat-4_*::*APOE*ε4, P*_unc-54_*::tdTomato])} in the absence of Aβ results in no difference from GFP expression only (*P*=0.5391, *P*=0.9823, *P*=0.8248, respectively). There was also no difference between *APOE*ε2 and *APOE*ε3 (*P*=0.8255), *APOE*ε2 and *APOE*ε4 (*P*=0.9824), or *APOE*ε3 and *APOE*ε4 (*P*=0.9825). *n*=90 for each strain; one-way ANOVA with Tukey's post hoc test. These data are reported as mean±s.e.m. All nematodes were grown at 20°C. (B) Animals expressing GFP alone display no neurodegeneration at days 3 or 7, in contrast to animals expressing Aβ that exhibit significant neurodegeneration at days 3 (*P*<0.0001) or 7 (*P*<0.0001). Co-expression of Aβ and *ApoE*ε2 {UA351[*baIn50*(P*_eat-4_*::*APOE*ε2, P*_unc-54_*::tdTomato); *baIn34*[P*_eat-4_*::Aβ,P*_myo-2_*::mCherry]; *adIs1240*(P*_eat-4_*::GFP)]} significantly attenuated neurodegeneration at days 3 (*P*=0.0397) and 7 (*P*=0.0002) post-hatching, whereas co-expression of *APOE*ε3 {UA353(*baIn51*[P*_eat-4_*::ApoEε3, P*_unc-54_*::tdTomato]; *baIn34*[P*_eat-4_*::Aβ,P*_myo-2_*::mCherry]; *adIs1240*[P*_eat-4_*::GFP])} resulted in no significant difference from Aβ alone at day 3 (*P*=0.02945). However, by day 7 post-hatching, co-expression of *ApoE*ε3 yielded a significant reduction in Aβ-mediated neurodegeneration (*P*=0.0102). In contrast, co-expression of *APOE*ε4 {UA355(*baIn52*[P*_eat-4_*::*APOE*ε4, P*_unc-54_*::tdTomato]; *baIn34*[P*_eat-4_*::Aβ,P*_myo-2_*::mCherry]; *adIs1240*[P*_eat-4_*::GFP])} resulted in no significant difference from Aβ alone at days 3 (*P*=0.9579) or 7 (*P*=0.9369) post-hatching. At day 3, there was no significant difference between Aβ+*APOE*ε2 and Aβ+*APOE*ε3 (*P*=0.5048), Aβ+ApoEε3 and Aβ+*APOE*ε4 (*P*=0.5225), or Aβ+*APOE*ε2 and Aβ+ApoEε4 (*P*=0.0797). However, at day 7 post-hatching, protection by *APOE*ε2 was significantly higher than that by *APOE*ε3 (*P*=0.028) and *APOE*ε4 (*P*=0.0001). Additionally, at day 7 post-hatching, protection by *APOE*ε3 was significantly higher than that by *APOE*ε4 (*P*=0.0049). *n*=90 for each strain; one-way ANOVA with Tukey's post hoc test. These data are reported as mean normalized to GFP animals±s.e.m. All nematodes were grown at 20°C. (C) Representative images of *C. elegans* glutamatergic tail neurons containing GFP (DA1240), Aβ alone (UA198), Aβ+*APOE*ε2 (UA351), Aβ+*APOE*ε3 (UA353) and Aβ+ApoEε4 (UA355). Arrows point to intact neurons, whereas arrowheads indicate sites of neurons that have degenerated. Scale bar: 10 µm. (D) Expression of Aβ (UA198) hampers mechanosensation (*P*<0.0001), but expression of the *APOE* alleles (UA356, UA357, UA358) alone, without Aβ co-expression, does not affect mechanosensory response (*P*>0.9999, *P*=0.9971, *P*>0.9999, respectively). Additionally, expression of the *APOE* alleles alone showed no statistically significant difference between *APOE*ε2 and *APOE*ε3 (*P*=0.9914), *APOE*ε2 and *APOE*ε4 (*P*=0.9994), or *APOE*ε3 and *APOE*ε4 (*P*=0.9994). The difference between Aβ-expressing animals and any of the *APOE* alleles alone was statistically significant (*P*<0.0001 in each comparison). *n*=90 for each strain; one-way ANOVA with Tukey's post hoc test. These data are reported as mean±s.e.m. (E) Glutamatergic expression of Aβ hampers the gentle touch response (*P*<0.0001). Aβ+*APOE*ε2 mitigates loss of mechanosensation (*P*=0.0095), but there was no significant difference between Aβ and either *APOE*ε3 (*P*=0.747) or *APOE*ε4 (*P*=0.644). Additionally, there was no significant difference between Aβ+*APOE*ε2 and Aβ+*APOE*ε3 (*P*=0.1429), Aβ+*APOE*ε2 and Aβ+*APOE*ε4 (*P*=0.1875), or Aβ+*APOE*ε3 and Aβ+*APOE*ε4 (*P*=0.9997). *n*=90 for each strain; one-way ANOVA with Tukey's post hoc test. These data are reported as mean±s.e.m. (F) Expression of *APOE* was determined by RT-qPCR of mRNA isolated from 100 animals for each of *APOE*ε2, *APOE*ε3 and *APOE*ε4. Amplification and Cq quantification by quantitative PCR shows twofold higher expression of *APOE*ε4 than *APOE*ε2 that is not statistically significant (*P*=0.2107). The fourfold higher expression of *APOE*ε3 than *APOE*ε2 was statistically significant (*P*=0.0127), but the difference between *APOE*ε3 and *APOE*ε4 was not statistically significant (*P*=0.1280). Values represent the mean±s.e.m. of three independent biological replicates each with three technical replicates; one-way ANOVA with Tukey's post hoc test. * denotes statistical significance; ns, nonsignificant.

To model ApoE activity in *C. elegans*, complementary DNAs (cDNAs) encoding the three distinct human *APOE* alleles (*APOE*ε2, *APOE*ε3 and *APOE*ε4) were recombined with the artificial constitutive *her-1* secretion signal, and expression was driven by the glutamatergic neuron-specific *eat-4* promoter. These three constructs were microinjected into wild-type (N2) animals, integrated into the genome and crossed with Aβ-expressing animals after outcrossing. Overexpression of Aβ induced neurodegeneration of glutamatergic neurons (Fig. 1A), as has been observed previously (Griffin et al., 2018; Tardiff et al., 2017; Treusch et al., 2011), while expression of *APOE*ε2, *APOE*ε3 or *APOE*ε4 in glutamatergic neurons did not impact neurodegeneration in the absence of Aβ (Fig. 1A).

Because the ε2 allele is associated with protective phenotypes (Bu, 2009; Liu et al., 2013a), we hypothesized that co-expression of *APOE*ε2 with Aβ would attenuate Aβ-induced neurodegeneration. At both days 3 and 7 post*-*hatching, nearly 100% of all animals expressing GFP alone have all five normal glutamatergic neurons. However, when co-expressed with Aβ, the *APOE*ε2 allele suppressed Aβ-mediated neurodegeneration by ∼30% at days 3 and 7 post-hatching (Fig. 1B,C). Furthermore, because the *APOE*ε3 allele appears functionally neutral in humans, and ε4 is associated with increased neurotoxicity (Bu, 2009; Corder et al., 1993; Huang and Mucke, 2012; Liu et al., 2013a), we hypothesized that *APOE*ε3 would elicit marginal or no neuroprotective effect, while *APOE*ε4 would increase neurodegeneration. At day 3, there was no statistically significant difference in neurodegeneration between animals expressing Aβ alone or co-expressing *APOE*ε3, but, at day 7, *APOE*ε3 significantly reduced Aβ-mediated neurodegeneration by ∼10%, which was significantly less than the protection afforded by *APOE*ε2. This protection was also significantly greater than that provided by the ApoEε4 strain, in which there was no change in neurodegeneration at either day 3 or day 7 (Fig. 1B,C). Although co-expression with *APOE*ε4 did not enhance neurodegeneration in this model, it was not statistically different from Aβ alone at days 3 or 7 (*P*=0.9579, *P*=0.9369, respectively), but was significantly different from the Aβ+ApoEε2 strain at day 7, thereby confirming earlier reports that there may be alternative mechanisms of action between these two alleles (Corder et al., 1994; Talbot et al., 1994).

As a secondary readout for glutamatergic neuronal dysfunction, we turned to a behavioral assay, as altered mechanosensory touch response is indicative of glutamatergic neuron dysfunction. In *C. elegans*, a pair of glutamatergic tail neurons have processes extending from the tail to the mid-body, to control forward escape in response to posterior gentle touch (Chalfie et al., 1985). In worms expressing Aβ in glutamatergic neurons, this posterior gentle touch response is defective (Fig. 1D). However, in worms expressing *APOE* alleles without Aβ, gentle touch response is not defective, indicating that the *APOE* alleles on their own are not pathogenic (Fig. 1D). When worms co-overexpressing Aβ and ApoEε2 were assayed in the touch response assay, there was a significant mitigation of this mechanosensory defect (Fig. 1E). Recovery was not observed by ApoEε3 or ApoEε4 co-expression since they were not significantly reduced compared with the Aβ+ApoEε2 strain (Fig. 1E). These data also suggest that, since there is a significant difference between Aβ+ApoEε2 and Aβ alone, but not between Aβ alone and either the Aβ+ApoEε3 or Aβ+ApoEε4 strains, there might be alternative mechanisms of action among these alleles that can be teased out using this assay. For example, although Aβ+ApoEε3 appeared to have a neuroprotective effect at later stages (day 7; Fig. 1B), the seemingly protected neurons in animals co-expressing ApoEε3 demonstrated reduced mechanosensory sensitivity. This suggests that ApoEε3 may confer moderate protection of neuronal structure that does not ameliorate loss of neuronal function by Aβ.

To ensure that the *APOE*-allele-specific phenotypes we observed are functionally driven and are not simply due to transgenic expression level differences, *APOE*ε2, *APOE*ε3 and *APOE*ε4 mRNA levels were quantified by reverse-transcription quantitative polymerase chain reaction (RT-qPCR: Fig. 1F). There were no statistically significant differences in relative normalized *APOE* transcripts between ApoEε2 and ApoEε4 samples (*P*=0.2107) or ApoEε3 and ApoEε4 samples (*P*=0.1280). However, *APOE*ε3 transcripts were significantly higher than *APOE*ε2 transcripts (*P*=0.0127). Taken together with the neurodegeneration analyses, these results indicate that ApoEε2 neuroprotection is likely not due to disproportionate overexpression compared with ApoEε3.

### *APOE*-allele-specific modulation of calcium homeostasis

**Fig. 2.**
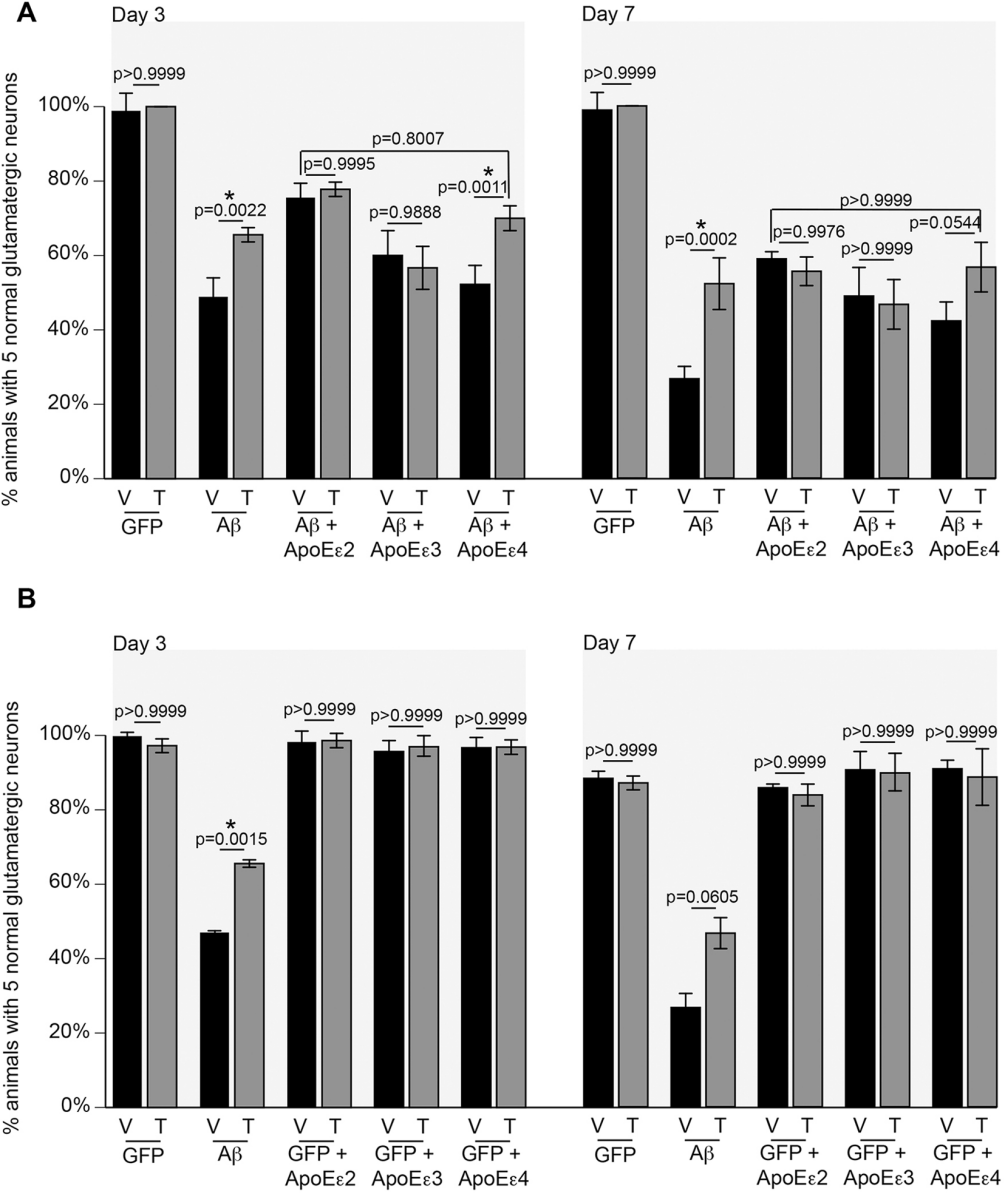
**Thapsigargin treatment reduces neurodegeneration with Aβ and Aβ+ApoEε4, but has no additive neuroprotective effect with either ApoEε2 or ApoEε3.** (A) At day 3 post-hatching, thapsigargin (T) has no effect on synchronized hermaphrodite populations expressing GFP alone in the glutamatergic neurons (DA1240; *P*>0.9999), but it attenuates neurodegeneration with Aβ compared with vehicle (V) control (UA198; *P*<0.0022). There was no observable difference between vehicle and thapsigargin treatments in Aβ+ApoEε2 (UA351; *P*=0.9995) or Aβ+ApoEε3 (UA353; *P*=0.9888). However, thapsigargin treatment reduced neurodegeneration in Aβ+ApoEε4 compared with vehicle (UA355; *P*=0.0011). This rescue was statistically insignificant when comparing Aβ+ApoEε2 with vehicle (*P*=0.8007). Similarly, at day 7 post-hatching, there was no difference between vehicle and thapsigargin treatments in animals expressing GFP alone (DA1240; *P*>0.9999), while thapsigargin reduced Aβ-mediated neurodegeneration (UA198; *P*<0.0002). Thapsigargin treatment had no effect on neurodegeneration in Aβ+ApoEε2 (UA351; *P*=0.9976) or Aβ+ApoEε3 (UA353; *P*>0.9999), and failed to attenuate neurodegeneration significantly with ApoEε4 co-expression (UA355; *P*=0.0544). When treated with thapsigargin, Aβ+ApoEε4 was not different from Aβ+ApoEε2 with vehicle (*P*>0.9999). *n*=90 for each strain; two-way ANOVA with Tukey's post hoc test. These data are reported as mean animals±s.d. All nematodes were grown at 20°C. (B) At days 3 and 7 post-hatching, thapsigargin had no effect on synchronized hermaphrodite populations expressing GFP alone in the glutamatergic neurons (DA1240; day 3, *P*>0.9999; day 7, *P*>0.9999), but thapsigargin (T) attenuates neurodegeneration with Aβ compared with vehicle (V) control (UA198; day 3, *P*<0.0015). The effect of thapsigargin on UA198 at day 7 was not statistically significant (*P*=0.0605). Without Aβ expression, thapsigargin has no statistically significant effect on neurodegeneration in ApoEε2 (UA356; day 3, *P*>0.9999; day 7, *P*>0.9999), ApoEε3 (UA357; day 3, *P*>0.9999; day 7, *P*>0.9999) or ApoEε4 animals (UA358; day 3, *P*>0.9999; day 7, *P*>0.9999). These data are reported as mean animals±s.d. *n*=90 for each strain; two-way ANOVA with Sidak's post hoc test. All nematodes were grown at 20°C. * denotes statistical significance.

To observe whether ApoE confers a physiologically relevant effect in our model, we examined the relationship between calcium homeostasis, Aβ and ApoE. In rat hippocampal neurons and chick sympathetic ganglia, ApoEε2 and ApoEε3 have no effect on *N*-methyl-D-aspartate (NMDA)-mediated calcium influx, but incubation with ApoEε4 results in massive NMDA-mediated calcium influx (Hartmann et al., 1994; Qiu et al., 2003; Tolar et al., 1999). In cultured mouse cortical neurons, the opposite effect is observed, wherein NMDA-mediated calcium influx is inhibited by ApoEε4 but exacerbated by ApoEε2 and ApoEε3 (Chen et al., 2010). Nevertheless, in both mammalian scenarios the functional impact of Aβ neurotoxicity was not assessed. To test the relationship between calcium, ApoE and Aβ in our model, we utilized thapsigargin, which increases cytosolic calcium concentrations by inhibiting the endoplasmic reticulum (ER) Ca^2+^-ATPase *sca-1*. Indeed, calcium influx induced by *APOE* has been partially attributed to ER calcium stores (Tolar et al., 1999). Animals expressing GFP alone were not impacted by thapsigargin treatment (Fig. 2A). Thapsigargin treatment of animals expressing Aβ attenuated neurodegeneration by nearly 20% compared with vehicle control at days 3 and 7 (Fig. 2A). There was no additive reduction in neurodegeneration by thapsigargin treatment with expression of either *APOE*ε2 or *APOE*ε3 at either day 3 or 7, suggesting that ApoE may potentially allay neurodegeneration in the same pathway as ER-derived calcium. As previously observed, the effect of ApoEε4 was significantly reduced when compared with ApoEε2 at both days 3 (*P*<0.0001) and 7 (*P*=0.0177), but together with thapsigargin, ApoEε4 showed protection similar to Aβ+ApoEε2 at both time points. No effect was observed from treatment of GFP animals expressing *APOE* without Aβ (Fig. 2B). These data suggest that ApoEε2 is neuroprotective through an interaction with ER-derived calcium and that this interaction is lost with the ApoEε4 protein variant.

**Fig. 3.**
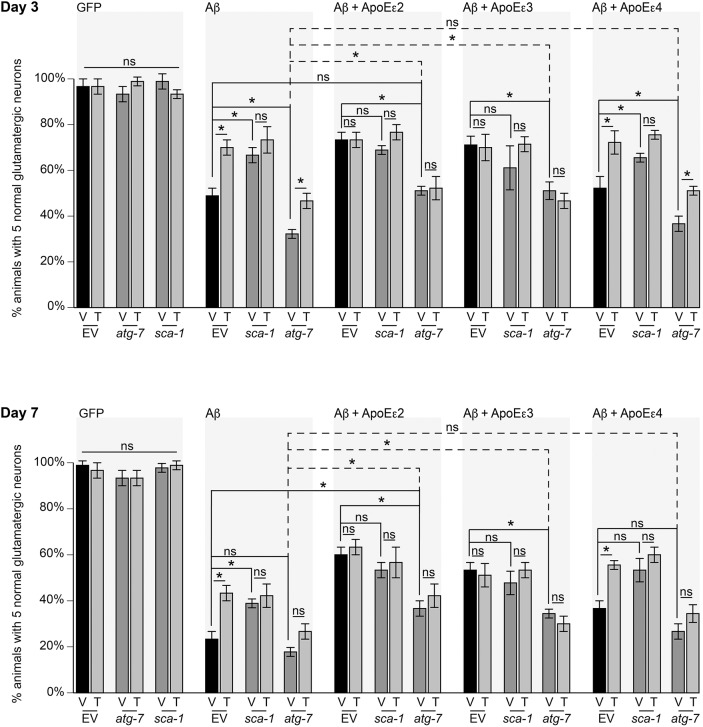
**Protection by thapsigargin is redundant with *sca-1*, but independent of *atg-7* in ApoEε2 and ApoEε3, but not ApoEε4, backgrounds.** To examine how thapsigargin impacts neurodegeneration we utilized a neuronal RNAi-sensitive strain crossed into the Aβ+ApoE backgrounds, in which we knocked down the ER Ca^2+^ ATPase homolog *sca-1*, or *atg-7*, required for the initiation of autophagy. These strains are designated as the following: Aβ glutamatergic-specific RNAi (no ApoE) {UA360(*sid-1*(*pk3321*); *baIn53*[P*_eat-4_*::*sid-1*, P*_dat-1_*::GFP]; *adIs1240*[P*_eat-4_*::GFP]; *baIn34*[P*_eat-4_*::Aβ,P*_myo-2_*::mCherry])}; Aβ+ApoEε2 glutamatergic-specific RNAi {UA364(*sid-1*(*pk3321*); *baIn53*[P*_eat-4_*::*sid-1*, P*_dat-1_*::GFP]; *adIs1240*[P*_eat-4_*::GFP]; *baIn50*[P*_eat-4_*::*APOE*ε2, P*_unc-54_*::tdTomato]; *baIn34*[P*_eat-4_*::Aβ,P*_myo-2_*::mCherry]}; Aβ+ApoEε3 glutamatergic-specific RNAi {UA365(*sid-1*(*pk3321*); *baIn53*[P*_eat-4_*::*sid-1*, P*_dat-1_*::GFP]; *adIs1240*[P*_eat-4_*::GFP]; *baIn51*[P*_eat-4_*::*APOE*ε3, P*_unc-54_*::tdTomato]; *baIn34*[P*_eat-4_*::Aβ,P*_myo-2_*::mCherry])} and Aβ+ApoEε4 glutamatergic-specific RNAi {UA366(*sid-1*(*pk3321*); *baIn53*[P*_eat-4_*::*sid-1*, P*_dat-1_*::GFP]; *adIs1240*[P*_eat-4_*::GFP]; *baIn52*[P*_eat-4_*::*APOE*ε4, P*_unc-54_*::tdTomato]; *baIn34*[P*_eat-4_*::Aβ,P*_myo-2_*::mCherry]}. At days 3 and 7 post*-*hatching, neither *atg-7* nor *sca-1* RNAi depletion had an effect on animals expressing GFP alone (UA359; *P*>0.9999 for each). Thapsigargin treatment (T) did not affect the phenotypes in the GFP background (UA359; *P*>0.9999 for each), but reduced Aβ-mediated neurodegeneration significantly at days 3 (UA360; *P*<0.0001) and 7 post-hatching (*P*<0.0001). Depletion of *sca-1* significantly reduced neurodegeneration at both days 3 (*P*=0.0002) and 7 (*P*=0.0009), but there was no statistically significant change when *sca-1*-depleted animals were treated with thapsigargin at either day 3 (*P*>0.9999) or 7 (*P*>0.9999). Although *atg-7* RNAi increased neurodegeneration in the Aβ background at day 3 (*P*=0.0007), the difference between empty vector (EV) and *atg-7* (RNAi) was not statistically significant at day 7 (*P*>0.9999). Similarly, thapsigargin was significantly protective with *atg-7* depletion at day 3 (*P*=0.0097), but not at day 7 (*P*=0.9111). As previously observed, thapsigargin treatment provided no additional protection with Aβ+*APOE*ε2 co-expression (day 3, *P*>0.9999; day 7, *P*>0.9999). There was no statistically significant difference in neurodegeneration between Aβ+ApoEε2 EV and *sca-1* RNAi (UA364; day 3, *P*>0.9999; day 7, *P*>0.9999) and no additional benefit of thapsigargin treatment with *sca-1* RNAi in the Aβ+ApoEε2 background (day 3, *P*=0.9992; day 7, *P*>0.9999). Depletion of *atg-7* in the Aβ+ApoEε2 background increased neurodegeneration at both days 3 (*P*<0.0001) and 7 (*P*<0.0001), but ApoEε2 still provided rescue with *atg-7* depletion when compared with Aβ alone with *atg-7* RNAi (day 3, *P*<0.0001; day 7, *P*=0.0007). Similar effects were observed in the Aβ+ApoEε3 background, including no additional protection with thapsigargin treatment compared with vehicle (V) (UA365; day 3, *P*>0.9999; day 7, *P*>0.9999), depletion of *sca-1* providing no additional protection with Aβ+ *APOE*ε3 co-expression (day 3, *P*=0.9368; day 7, *P*>0.9999), and no additive protection with thapsigargin treatment and *sca-1* RNAi (day 3, *P*=0.5193; day 7, *P*>0.9999). Similarly, *atg-7* RNAi significantly increased neurodegeneration compared with EV control (day 3, *P*<0.0001; day 7, *P*<0.0001), but it was still statistically significantly neuroprotective compared with Aβ alone with *atg-7* RNAi (day 3, *P*<0.0001; day 7, *P*=0.0002). Thapsigargin treatment did not reduce neurodegeneration with *atg-7* RNAi in the Aβ+ApoEε3 background (day 3, *P*>0.9999; day 7, *P*>0.9999). In contrast, as previously observed, thapsigargin was protective in the Aβ+ApoEε4 background at both days 3 (UA366; *P*<0.0001) and 7 (*P*<0.0001). Although *sca-1* RNAi conferred no additional protection with co-expression of Aβ+*APOE*ε2 or Aβ+*APOE*ε3, *sca-1* RNAi reduced neurodegeneration in the Aβ+ApoEε4 background (day 3, *P*=0.0330; day 7, *P*=0.0330). Again, thapsigargin treatment did not decrease neurodegeneration in the Aβ+ApoEε4 background with *sca-1* RNAi (day 3, *P*=0.6339; day 7, *P*>0.9999). Depletion of *atg-7* significantly increased neurodegeneration in the Aβ+ApoEε4 background at day 3 (*P*=0.0027), but it did not significantly increase neurodegeneration at day 7 (*P*=0.4216). Expression of ApoEε4 conferred no significant protection against Aβ-mediated neurodegeneration with *atg-7* RNAi when compared with Aβ alone (day 3, *P*>0.9999; day 7, *P*=0.8111). In contrast to the Aβ+ApoEε2 or Aβ+ApoEε3 backgrounds, thapsigargin provided significant protection with depletion of *atg-7* in the Aβ+ApoEε4 background at day 3 (*P*=0.0097), but there was no statistically significant difference induced by thapsigargin with *atg-7* RNAi at day 7 (*P*=0.9909). *n*=90 for each line; * indicates statistical significance; ns, not significant; two-way ANOVA with Tukey's post hoc test. These data are reported as mean±s.d. All nematodes were grown at 20°C.

To confirm that the observed effect by thapsigargin is related to its inhibition of *sca-1*, a Ca^2+^ ATPase and target of thapsigargin, we generated a conditional RNAi-sensitive strain, in which RNAi is restricted to the glutamatergic neurons. This strain was then crossed into the Aβ and Aβ+ApoE backgrounds, so that genetic targets can be depleted with co-expression of Aβ and ApoE (Table 1). As previously observed, thapsigargin treatment reduced neurodegeneration in animals expressing Aβ alone and co-expressing Aβ+ApoEε4, but not in either Aβ+ApoEε2 or Aβ+ApoEε3 animals (Fig. 3). Depletion of *sca-1* in Aβ alone was neuroprotective when compared with empty vector (EV) control, but there was no additional protection conferred by a combination of *sca-1* RNAi and thapsigargin treatment, suggesting that protection by thapsigargin, redundant with ApoEε2 and ApoEε3, is not independent from its target, *sca-1*. In contrast, *sca-1* RNAi was protective in the backgrounds expressing Aβ alone and Aβ+*APOE*ε4. Taken together, these data suggest that there is a genetic relationship between *APOE*ε2 and *sca-1* that is lost in the *APOE*ε4 genetic background.

**Table 1.**
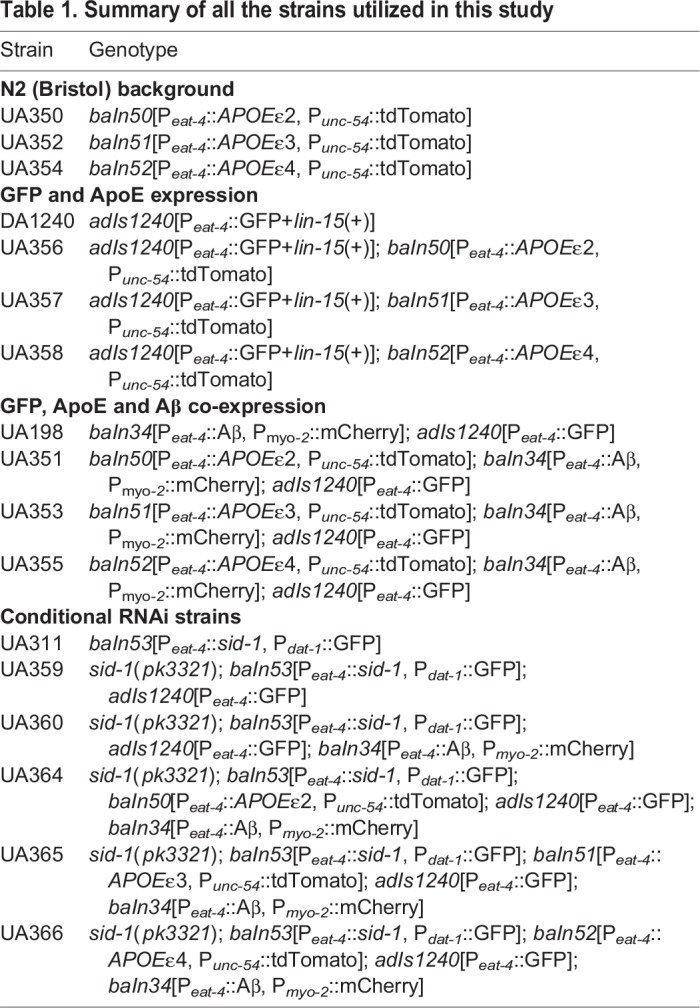
**Summary of all the strains utilized** **in this study**

Thapsigargin-induced alterations in ER-derived Ca^2+^ dynamics have been reported to also increase autophagy (Høyer-Hansen et al., 2007). Conversely, thapsigargin has also been observed to block degradation of autophagosomes without altering basal autophagy or maturation of autophagosomes (Ganley et al., 2011). To examine the relationship between thapsigargin, autophagy, Aβ and ApoE, neurodegeneration was examined in the conditional RNAi-sensitive strains with depletion of *atg-7*, which is required for the initiation of autophagy. Depletion of *atg-7* increased neurodegeneration in animals expressing Aβ alone (Fig. 3), but the difference was no longer statistically significant by day 7 (Fig. 3). With *atg-7* RNAi, thapsigargin treatment was significantly protective (Fig. 3) until day 7 (Fig. 3). Depletion of *atg-7* also increased neurodegeneration in both Aβ+ApoEε2 and Aβ+ApoEε3 backgrounds, but with significantly less degeneration than Aβ alone with *atg-7* RNAi, suggesting that protection by ApoEε2 and ApoEε3 is independent of autophagy. There was also no additional protection afforded by thapsigargin in the Aβ+ApoEε2 or Aβ+ApoEε3 backgrounds with *atg-7* RNAi, further indicating that ApoEε2 and ApoEε3 participate with calcium homeostasis to mediate protection. In contrast, *atg-7* RNAi increased neurodegeneration in the Aβ+ApoEε4 background, but was attenuated with thapsigargin treatment, further revealing the dysfunctional relationship between ApoEε4 and calcium homeostasis.

### Attenuation of neurodegeneration by starvation is independent of ApoE function

**Fig. 4.**
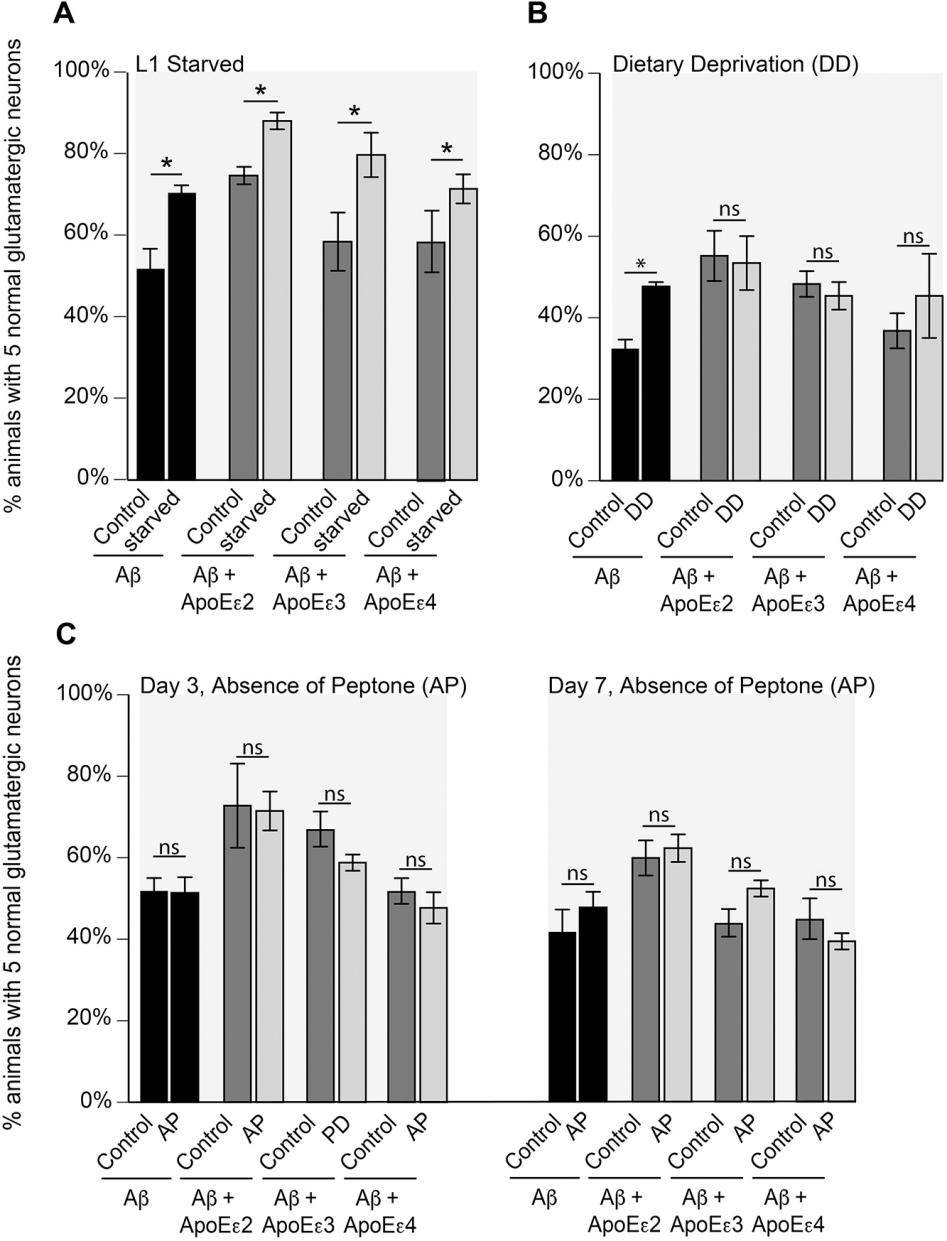
**Starvation of animals during the L1 larval stage attenuates neurodegeneration in all transgenic strains.** (A) Synchronized animals were deprived of food during the L1 stage and then assayed for neurodegeneration as young adults at day 3 post-hatching. Animals expressing Aβ (UA198; *P*=0.001) exhibited reduced neurodegeneration. Additionally, animals expressing Aβ+*APOE*ε2 (UA351; *P*=0.0156), Aβ+*APOE*ε3 (UA353; *P*=0.0003) or Aβ+*APOE*ε4 (UA355; *P*=0.0201) also displayed reduced neurodegeneration when deprived of food as L1 larvae. *n*=90 for each line; two-way ANOVA with Sidak's post hoc test. These data are reported as mean normalized to GFP animals±s.d. All nematodes were grown at 20°C. (B) On the second day of adulthood (day 5 post-hatching), animals were moved to plates absent of bacteria, according to the dietary-deprivation regimen outlined by Greer and Brunet (2009), which reported that dietary-deprivation-dependent lifespan extension was dependent on *hsf-1*. Neurodegeneration analysis of dietary-deprived animals was performed on day 7 post-hatching. Dietary deprivation reduced neurodegeneration in animals expressing Aβ alone (UA198; *P*=0.0107), but there was not a significantly additive effect in either Aβ+ApoEε2 (UA351; *P*=0.9909), Aβ+ApoEε3 (UA353; *P*=0.9441) or Aβ+ApoEε4 (UA355; *P*=0.2421) *C. elegans*. These data are reported as mean normalized to GFP animals±s.d. *n*=90 for each line; * indicates statistical significance; ns, not significant; two-way ANOVA with Sidak's post hoc test. All nematodes were grown at 20°C. (C) The absence of peptone has been reported to increase lifespan through the AMP-activated protein kinase, AAK-2, and the insulin-like signaling protein, DAF-16. Synchronized animals were grown at 20°C on either NGM with a standard final peptone concentration of 2.5 g/l or NGM without peptone and analyzed at days 3 and 7 post-hatching. Absence of peptone had no effect on Aβ alone (UA198; day 3, *P*=0.9998; day 7, *P*=0.2740), Aβ+ApoEε2 (UA351; day 3, *P*=0.9965; day 7, *P*=0.9230), Aβ+ApoEε3 (UA353; day 3, *P*=0.2798; day 7, *P*=0.0653) or Aβ+ApoEε4 (UA355; day 3, *P*=0.8268; day 7, *P*=0.3279). These data are reported as mean normalized to GFP animals±s.d. *n*=90 for each line; * indicates statistical significance; ns, not significant; two-way ANOVA with Sidak's post hoc test.

Starvation and caloric restriction increase health and lifespan through multiple pathways that overlap with significant conservation among yeast, *C. elegans*, *Drosophila*, rodents and primates (Fontana et al., 2010). Furthermore, dietary restriction reduces Aβ toxicity (Steinkraus et al., 2008). We therefore hypothesized that starvation would attenuate Aβ-mediated neurodegeneration and tested its effect in the context of the three distinct *APOE* alleles. To test this, synchronized embryos were hatched onto unseeded plates and incubated for 24 h, after which time they were transferred to normal (nematode growth medium; NGM) nematode plates seeded with *Escherichia* *coli*. Although early-L1-stage larval starvation attenuated neurodegeneration as expected in worms expressing Aβ alone, this protective effect was also shared indiscriminately with animals co-expressing any of the *APOE* alleles (Fig. 4A). These data suggest that, in modulating its effects on neuron survival, ApoE operates outside of this starvation-induced rescue response, thus excluding this mechanism of dietary restriction as an *APOE*-allele-specific means of modulating neurotoxicity. However, alternative dietary restriction regimens in *C. elegans* have been found to extend lifespan through parallel or overlapping pathways (Greer and Brunet, 2009). The extension of lifespan by dietary deprivation was dependent on heat shock factor 1 (*hsf-1*), while AMP-activated protein kinase 2 (*aak-2*) and FOXO/*daf-16* were required for lifespan extension by the absence of peptone. Because the dietary deprivation regimen begins dietary restriction at day 2 of adulthood (day 5 post-hatching), animals were washed off food at day 5 post-hatching and moved to unseeded plates until analysis at day 7. Although dietary deprivation reduced neurodegeneration in the background expressing Aβ alone (Fig. 4B), dietary deprivation provided no statistically significant rescue in the Aβ+ApoEε2, Aβ+ApoEε3 or Aβ+ApoEε4 backgrounds, suggesting that the ApoE protein, irrespective of allelic variation, might generally interfere with *hsf-1*-associated protective mechanisms. In contrast, there was no statistically significant change in neurodegeneration in animals subjected to the absence of peptone regimen at either days 3 or 7 (Fig. 4C).

### Survival shortened by Aβ is rescued by ApoEε2 and ApoEε3, but not ApoEε4

**Fig. 5.**
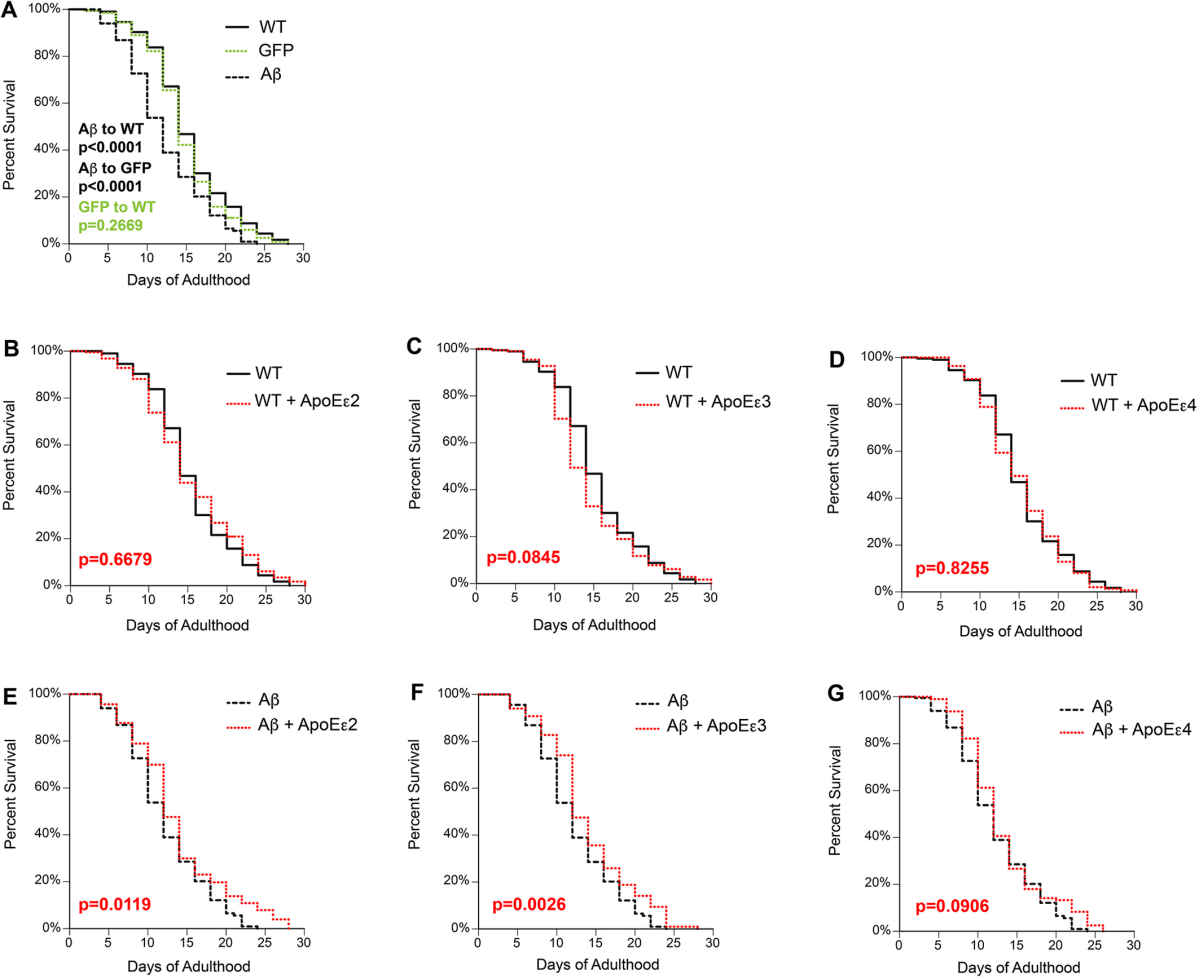
**Effects of apolipoprotein E isoforms and Aβ expression on *C. elegans* survival.** Animal populations were synchronized by a 2-h egg lay and maintained at 20°C. The L4 molt was defined as *t*=0, and survival was assessed by scoring response to mechanical probing. (A) Aβ expression (UA198) significantly reduced survival, compared with the survival curves for both wild-type (WT) N2 nematodes (*P*<0.0001) and nematodes expressing GFP alone in glutamatergic neurons (DA1240; *P*<0.0001). There was no significant difference between N2 and expression of GFP alone (DA1240; *P*=0.2669). (B-D) Survival curves comparing N2 with ApoEε2 {UA350 (*baIn50*[P*_eat-4_*::*APOE*ε2, P*_unc-54_*::tdTomato])} (B), ApoEε3 {UA352 (*baIn50*[P*_eat-4_*::*APOE*ε3, P*_unc-54_*::tdTomato])} (C) and ApoEε4 {UA354 (*baIn50*[P*_eat-4_*::*APOE*ε4, P*_unc-54_*::tdTomato])} (D). Survival curves were not significantly different between N2 and ApoEε2 (UA350; *P*=0.6679), N2 and ApoEε3 (UA352; *P*=0.0845), or N2 and ApoEε4 (UA354; *P*=0.8255). (E-G) Survival curves comparing Aβ with Aβ+ApoEε2 (E), Aβ+ApoEε3 (F) and Aβ+ApoEε4 (G). (E) The presence of ApoEε2 with Aβ (UA351) significantly increases survival compared with Aβ alone (*P*=0.0119). (F) Survival was also significantly increased in Aβ+ApoEε3 (UA353) compared with Aβ alone (*P*=0.0026). (G) In contrast, Aβ+ApoEε4 (UA355) did not significantly alter survival (*P*=0.0906). *n*=200 for each line. The log-rank (Mantel–Cox) method to account for differences in survival was applied for statistical analysis of all strains.

Because AD is an age-related disease and *APOE*ε4 homozygosity is associated with earlier onset of AD (Bu, 2009; Corder et al., 1993; Liu et al., 2013a), we examined how the relationship between Aβ and ApoE in the glutamatergic neurons affected survival with aging. Additionally, the Mantel–Cox/log-rank method was used for survival analyses, as it assigns equal weights in statistical calculations for the entire pattern or path of the curve, not just the median or maximum values displayed. Both wild type (Bristol N2) and animals expressing GFP alone exhibited similar survival curves that were not significantly different from each other (Fig. 5A). In animals expressing Aβ, survival was significantly reduced (Fig. 5A), suggesting a relationship between glutamatergic neurodegeneration and aging in the *C. elegans* neuronal model. In animals expressing *APOE* alleles alone (encoding ApoEε2, ApoEε3 or ApoEε4), the survival curves were similar to the N2 control (Fig. 5B-D). However, co-expression of Aβ+ApoEε2 or Aβ+ApoEε3 increased survival (Fig. 5E,F), compared with Aβ alone (Fig. 5A). In contrast, co-expression of Aβ+ApoEε4 had no significant effect compared with Aβ alone (Fig. 5G). These data suggest that integrity of the glutamatergic neurons through the aging process, as differentially modulated by the *APOE* alleles in the presence of Aβ, affects whole-animal survival.

## DISCUSSION

The *APOE*ε4 allele is the strongest risk factor associated with late-onset AD, yet determining precisely how the *APOE* alleles differentially modulate Aβ toxicity and neuronal behavior remains unresolved. An expedient examination of the relationship between the *APOE* alleles and Aβ requires a model system in which neuronal dysfunction and loss are amenable to both genetics and tractable neuronal outputs. Our *C. elegans* model of Aβ-induced neurodegeneration in glutamatergic neurons recapitulates mammalian and cell culture models for AD-associated gene analyses (Griffin et al., 2017; Matlack et al., 2014; Treusch et al., 2011). Furthermore, genes associated with AD have *C. elegans* orthologs (Mukherjee et al., 2017; Vahdati Nia et al., 2017). Here, we debut a model of ApoE activity in our established neuronal *C. elegans* background and suggest that it can be exploited to examine the relationship between ApoE and Aβ for neuronal behavior, integrity and proteotoxicity.

Mammalian and cell culture models show that the *APOE*ε2 allele provides a protective effect against Aβ-mediated neurodegeneration, while the most prevalent allele, *APOE*ε3, provides none (Bu, 2009; Corder et al., 1993; Huang and Mucke, 2012; Liu et al., 2013a). *APOE*ε4 is associated with enhanced susceptibly and earlier onset of AD, as well as exacerbated neurodegeneration. Studies have shown that the *APOE*ε2 allele may be neuroprotective through a mechanism that consists of more than simply the absence of the *APOE*ε4 allele (Corder et al., 1994; Talbot et al., 1994). It is noteworthy that in several of our assays, we identified contrasting phenotypes from *C. elegans* with either Aβ+ApoEε2 or Aβ+ApoEε4. As an illustration, overexpression of human ApoEε2 in *C. elegans* vitiates Aβ-mediated neurodegeneration, whereas ApoEε3 only appears to have a rescuing phenotype later in life (Fig. 1B). However, the neuroprotective effect observed was not recapitulated by the ApoEε4 variant (Fig. 1B). These data, which are functionally reflective of the well-established clinical susceptibility profile associated with ApoE, highlight the conservation of the neurodegenerative consequences that arise with the allelic distribution associated with AD. While loss of neuroprotective function in the Aβ+ApoEε4 background represents a mechanistically relevant observation, additional avenues of ApoEε4-associated alterations in cell biology remain to be explored. For example, although the ε4 allele is typically associated with increased Aβ toxicity and disruption of homeostatic pathways *per se*, we observe no increase in neurodegeneration by the *APOE*ε4 allele. This may be due to a C-terminal proteolytic product of *APOE*ε4 that more strongly induces cellular responses associated with neurodegeneration (Bien-Ly et al., 2011; Brecht et al., 2004; Harris et al., 2003; Tolar et al., 1999). However, the effectors of this cleavage are unknown. Yet, full-length ApoEε4 has been observed to alter expression of sirtuin, which could affect observable phenotypes under additional stress (Lattanzio et al., 2014; Theendakara et al., 2013, 2016). Thus, *C. elegans* might be an effective model for examining how full-length ApoEε4 and its truncate modify Aβ toxicity *in vivo*.

Calcium homeostasis is found to be perturbed in AD, particularly by ApoE through glutamatergic (NMDA) receptor function (Chen et al., 2010; Hartmann et al., 1994; Qiu et al., 2003; Tolar et al., 1999). Thapsigargin treatment increases cytosolic calcium levels by inhibiting calcium uptake into the ER and we find that it mitigates Aβ toxicity, but not in the presence of either ApoEε2 or ApoEε3 (Fig. 2A), suggesting that ApoE has a function within calcium homeostasis that is selectively lost by the *APOE*ε4 allele. Whether this is dependent on glutamatergic receptors in our model is not yet clear. However, it appears that ER-derived calcium also contributes to ApoEε4-associated calcium defects (Tolar et al., 1999). Notably, autophagy has also been shown to be impaired in AD. Although it may be induced by thapsigargin treatment, these data suggest that autophagy and ApoE participate with Aβ toxicity through separate mechanisms. Despite this, the relationship between autophagy and calcium is not entirely clear (Sun et al., 2016). Future analyses using the Aβ+ApoE transgenic worm models could include autophagy, as its component proteins are highly conserved in *C. elegans* (Chang et al., 2017; Martinez et al., 2015; Stavoe et al., 2016).

Induction of autophagy by thapsigargin is reported to occur through stimulation of ER stress (Bernales et al., 2006; Ding et al., 2007; Høyer-Hansen et al., 2007; Kouroku et al., 2007). Although our data suggest divergent participation in protection between autophagy and ApoE, they do not preclude the possibility of ER stress. Whether ApoEε2 yields protection by inducing ER stress is unclear. It is, however, unlikely, considering that ApoEε4 has been shown to significantly increase ER stress compared with ApoEε3 in mice (Verghese et al., 2013; Zhong et al., 2009). In such a paradigm, increased ER stress by ApoEε2 would presumably recapitulate ApoEε4-associated phenotypes. Further, the protective effect of ApoEε2 might not be attributed to differences in ER stress induction, as ApoEε2 and ApoEε3 have been reported to have no difference in the expression of ER stress targets IRE1 (also known as ERN1), BiP (also known as HSPA5) and CHOP (also known as DDIT3), which increase, instead, with ApoEε4 expression (Verghese et al., 2013). Rather, stress and injury typically increase the expression of ApoE in brains (Xu et al., 2006). The effect of increased ApoE expression during stress might be due to mitochondrial interactions, because RNA sequencing of mouse brains revealed *Apoe*-allele-specific responses in mitochondrial gene expression (Babenko et al., 2017; Xu et al., 2006). Indeed, ApoEε3 is less likely to be retained at the ER (Brodbeck et al., 2011), and although the retention of ApoEε2 in the ER has not been reported, the effect of ER retention is due to the S61R present in ApoEε3 and ApoEε4, but not present in ApoEε2, thus making ApoEε2 far less likely to be retained at the ER. Perhaps the additional cysteine residues in ApoEε2 compared with ApoEε3 or ApoEε4 make ApoEε2 an agent of redox stabilization at mitochondria during stress (Yamauchi et al., 2017). Additionally, variations in the translocase of outer mitochondrial membrane 40 (TOMM40) and ApoE are associated with differences in longevity (Lin et al., 2016). Notwithstanding, the interaction between ApoE and the ER stress pathway is poorly understood and deserves to be more explicitly delineated.

Yeast and mammalian models have provided insights into the relationship between neurodegenerative disease, calcium and mitochondria that have been further recapitulated in *C. elegans* (Bornhorst et al., 2014; Caraveo et al., 2014; Kim et al., 2018; Martinez et al., 2017b; Ray et al., 2014). Given the decline of the mitochondrial unfolded protein response (UPR^mt^) with aging (Baker and Haynes, 2011), and that ApoEε4 increases activity at the mitochondria-associated membrane (MAM) (Tambini et al., 2016), the interaction between ApoEε2 and calcium may stabilize the relationship between the ER and the mitochondrion that is otherwise disrupted by Aβ and exacerbated by ApoEε4. Fusion, fission and recycling of mitochondria are largely affected by their association with the ER, and ApoE has been observed to elute with MAM fractions (Rusiñol et al., 1994). It is through these MAMs that calcium is transferred between the mitochondrion and ER to regulate cell death and metabolism (Marchi et al., 2018; Patergnani et al., 2011). ApoEε4, but not ApoEε3, upregulates MAM activity by increasing communication and facilitating function between the ER and the mitochondrion (Tambini et al., 2016). Alterations in the UPR^mt^ significantly affected Aβ toxicity in mouse, cell culture and *C. elegans* (Sorrentino et al., 2017). Treatment with doxycycline was found to increase the UPR^mt^ through *atfs-1* and reduce Aβ-associated deficits in a *C. elegans* model of Aβ expression in muscles. Indeed, mitochondria secrete a peptide, humanin, under stress conditions to modulate MAMs and protect against cell death (Sreekumar et al., 2017). Supplementation of a humanin derivative reduced cognitive defects in a transgenic AD mouse model (Niikura et al., 2011). Future work would include measuring changes in intracellular calcium with expression of the different *APOE* alleles, to determine whether these changes are dependent on the mitochondrial calcium uniporter or ER stress, and how these affect mitochondrial stability towards neuronal integrity. As such, this model provides a potent medium with which to further understand and probe these interactions for therapeutic targets.

Loss or depletion of the insulin signaling receptor, *daf-2*, doubles lifespan in *C. elegans* (Kenyon et al., 1993) in a manner that is independent of autophagy (Greer and Brunet, 2009). In *C. elegans* models of proteotoxicity, loss of *daf-2* reduces α-synuclein-mediated neurodegeneration (Knight et al., 2014; Ray et al., 2014), paralysis-induced poly-Q toxicity (Steinkraus et al., 2008) and paralysis-induced Aβ toxicity (Cohen et al., 2006; Florez-McClure et al., 2007; Steinkraus et al., 2008). Furthermore, loss of *daf-2* decreases Aβ toxicity (Steinkraus et al., 2008) by increasing the autophagic clearance of Aβ (Florez-McClure et al., 2007). However, different longevity association pathways are activated in response to diverse dietary restriction regimens (Greer and Brunet, 2009). Peptone absence extends lifespan through *aak-2* and FOXO/*daf-16*, but yielded no change in neurodegeneration, suggesting that *daf-2*-mediated protection observed in other Aβ models (Cohen et al., 2006) might be engaged through downstream mechanisms in parallel with AAK-2 activation of FOXO/*daf-16.* For example, reduced insulin-like signaling decreased Aβ accumulation by elevating autophagy and lysosome populations (Florez-McClure et al., 2007). In the dietary deprivation model, the extension of lifespan requires *hsf-1*. In such a model, it is feasible that the complete absence of food during the first larval stage could activate responses controlled by HSF-1 activity independently of expression of any *APOE* allele (Steinkraus et al., 2008). However, this would stand in opposition to the *hsf-1*-dependent dietary deprivation model that begins starvation 2 days into adulthood (Greer and Brunet, 2009; Steinkraus et al., 2008), but was only protective with Aβ alone (Fig. 4B), suggesting an interaction between ApoE in the dietary deprivation model that does not take place in the L1 starvation model. We show a potent neuroprotective effect of two different dietary restriction regimens that interact differently with ApoE in Aβ toxicity *in vivo* (Fig. 4A,B). Considering the robust understanding and utility of *C. elegans* in aging research, this model opens avenues for more thorough examination of the relationships between longevity pathways, ApoE and Aβ*.*

Although ApoE is associated with longevity (Fuku et al., 2017; Lin et al., 2016; Schächter et al., 1994; Skillbäck et al., 2018), it is not clear how ApoE interacts with other longevity-associated pathways, especially when challenged by Aβ-induced proteostatic stress. A more thorough understanding of transcriptional changes with ApoE expression would shed light on the neuronal effect of ApoE that drives the differences between L1 starvation and dietary deprivation models. ApoEε4 has been observed to translocate to the nucleus and alter gene expression by altering transcriptional regulation (Lattanzio et al., 2014; Theendakara et al., 2013, 2016). Many of these genes appear conserved from *C. elegans* to humans and might have similar implications for metabolism, stress response and aging (Arey and Murphy, 2017; Vahdati Nia et al., 2017). Thus, future studies combining transcriptional profiling of the ApoE-Aβ transgenics with RNAi or genetic depletion of up- or downregulated target genes would be informative.

Aging remains the most definitive risk factor for AD. Therefore, it is significant to note that, in the absence Aβ, none of the *APOE* alleles had an effect on survival (Fig. 5B-D). However, when independently co-expressed with Aβ, both *APOE*ε2 and *APOE*ε3 attenuated the shortened survival caused by Aβ (Fig. 5A,E,F). Although the observed differences between the survival curves were modest, they were statistically significant. In contrast, *APOE*ε4 did not confer any significant effect (Fig. 5G). One possibility to explain these results is that the shortened survival induced by Aβ (Fig. 5A) is a consequence of glutamatergic neuron failure to accurately control feeding behaviors and fat storage (Chun et al., 2015; Hamilton et al., 2005; Lee and Ashrafi, 2008; Greer and Brunet, 2009; Greer et al., 2008; Hills et al., 2004; Kindt et al., 2007; Lee et al., 2008; Zheng et al., 1999). Because the glutamatergic neuronal circuitry modulates feeding behaviors, Aβ might possibly depress survival through dysfunctional feeding, which is hitherto repressed by the protection of neuronal structure observed with *APOE*ε2 co-expression. The ability of the *APOE*ε2 allele to reduce survival depression by Aβ would therefore be due to restored glutamatergic connectivity through the associated neuroprotective phenotypes. Uninhibited feeding, in combination with the utilization of fat storage from loss of glutamatergic signaling, potentially incites insulin signaling responses that influence longevity (Greer et al., 2008; Gusarov et al., 2017). The connection between insulin signaling and longevity was first realized in *C. elegans* (Kenyon et al., 1993). Clinical research shows a complex relationship between diabetes, AD and ApoEε4 (Arnold et al., 2018). Administration of insulin facilitated memory recall in patients carrying *APOE*ε2 or *APOE*ε3, but further impaired recollection in *APOE*ε4 patients (Reger et al., 2006). Considering the history and utility of *C. elegans* in the study of aging, we propose this model would be an effective tool to study the relationship between aging, insulin signaling and ApoE variants in Aβ-induced neurodegeneration.

It should be noted that known functions of ApoE are not limited to the nervous system (McNeill et al., 2010; Rosenson et al., 2017; Zhang et al., 2010). By restricting the expression of alleles to a single cell type, as in our model, the cellular and subcellular effects can be isolated from the emergent effects of endogenous expression that would otherwise compound the complexity underlying Aβ-mediated neurodegeneration. Furthermore, that *C. elegans* has no endogenous ApoE ortholog allows use of this model for dissection of the interactions between ApoE and evolutionarily conserved pathways without obfuscation from other perturbations, such as immunological and hepatic responses typically associated with ApoE. Because of the genetic and pharmacological amenability of *C. elegans*, screening for modifiers of ApoE-Aβ activity is tenable. Additional phenotypic outputs might provide further insight into nuances of ApoE-induced effects. Because the glutamatergic signaling that regulates fat storage in response to food also modulates pharyngeal pumping rate (Greer and Brunet, 2009), both fat storage (Yen et al., 2010) and pharyngeal pumping (Sanders et al., 2017) are potential quantifiable outputs of glutamatergic signaling. Likewise, the olfactory circuit is modulated by glutamatergic signaling (Chalasani et al., 2007), exhibiting quantifiable changes in turning and reversals (Bhattacharya et al., 2014; Xiao et al., 2015) in response to specific odors (Chalasani et al., 2010). Furthermore, the *C. elegans* olfactory circuitry is a workshop for research in the neurobiological basis of learning (Cho et al., 2016). Consequently, candidate compounds can be tested for their effects on neurodegeneration, and also how they affect neuron function and animal health. Thus, this model provides a new medium through which neuronal mechanisms of ApoE can be distinctly probed to expedite the identification of therapeutic targets and risk factors to better address the urgent and unmet societal burden represented by AD.

## MATERIALS AND METHODS

### Plasmid construction

The cDNAs of the human *APOE* alleles were a generous gift from Susan Lindquist. The cDNAs were cloned by Gateway Technology (Invitrogen) according to the manufacturer’s protocol. Briefly, primers 5′-GGGGACAAGTTTGTACAAAAAAGCAGGCTCCatgcataaggttttgctggcactgttctttatctttctggcaccagcaATGaaggtggagcaagcggtgg-3′ and 5′-ggggaccactttgtacaagaaagctgggtcCTAcagtgattgtcgctgggcac-3′ were used to amplify the *APOE* alleles and amplica were recombined with pDONR221 by BP reaction to generate entry clones. Entry clones were confirmed by sequencing and recombined with P*_eat-4_* expression vectors by LR reaction. Expression clones were confirmed by sequencing.

### *C. elegans* strains

*C. elegans* were maintained following standard procedures (Brenner, 1974). To generate the worm ApoE models (Table 1), expression constructs were injected into Bristol N2 animals at 50 ng/μl with the co-injection marker transgene (P*_unc-54_*::tdTomato) at 50 ng/μl. At least three stable independent lines were generated, crossed with UA198 (*baIn34*[P*_eat-4_*::Aβ, P*_myo-2_*::mCherry]; *adIs1240*[P*_eat-4_*::GFP]) and analyzed for each *C. elegans* transgenic construct. Representative transgenic lines were selected and the corresponding transgenic lines in the N2 background were integrated using a Spectrolinker XL-1500 (Spectronics Corporation, Westbury, NY, USA). Integrated strains were outcrossed three times to N2 worms to generate the following strains: UA350 (*baIn50*[P*_eat-4_*::*APOE*ε2, P*_unc-54_*::tdTomato]), UA352 (*baIn51*[P*_eat-4_*::*APOE*ε3, P*_unc-54_*::tdTomato]) and UA354 (*baIn52*[P*_eat-4_*::*APOE*ε4, P*_unc-54_*::tdTomato]) (Table 1). These were crossed with UA198 to generate the following strains: UA351 (*baIn50*[P*_eat-4_*::*APOE*ε2, P*_unc-54_*::tdTomato]; *baIn34*[P*_eat-4_*::Aβ,P*_myo-2_*::mCherry]; *adIs1240*[P*_eat-4_*::GFP]), UA353 (*baIn51*[P*_eat-4_*::*APOE*ε3, P*_unc-54_*::tdTomato]; *baIn34*[P*_eat-4_*::Aβ, P*_myo-2_*::mCherry]; *adIs1240*[P*_eat-4_*::GFP]) and UA355 (*baIn52*[P*_eat-4_*::*APOE*ε4, P*_unc-54_*::tdTomato]; *baIn34*[P*_eat-4_*::Aβ, P*_myo-2_*::mCherry]; *adIs1240*[P*_eat-4_*::GFP]) (Table 1). They were also crossed with DA1240 to generate strains UA356 {*adIs1240*[P*_eat-4_*::GFP+lin-15(+)]; *baIn50*[P*_eat-4_*::*APOE*ε2, P*_unc-54_*::tdTomato]}, UA357 {*adIs1240*[P*_eat-4_*::GFP+*lin-15*(+)]; *baIn50*[P*_eat-4_*::*APOE*ε3, P*_unc-54_*::tdTomato]} and UA358 {*adIs1240*[P*_eat-4_*::GFP+*lin-15*(+)]; *baIn50*[P*_eat-4_*::*APOE*ε4, P*_unc-54_*::tdTomato]}.

To generate conditional RNAi-sensitive strains, N2 animals were injected with the glutamatergic neuron promoter-*sid-1* construct (P*_eat-4_*::*sid-1*) with a co-injection marker (P*_dat-1_*::GFP), integrated and outcrossed as previously described, to produce strain UA311 (baIn53[P*_eat-4_*::si*d*-1, P*_dat-1_*::GFP]). This strain was then crossed with the *sid-1(pk3321)* mutant to generate strain UA359 {*sid-1*(*pk3321*); *baIn53*[P*_eat-4_*::*sid-1*, P*_dat-1_*::GFP]; *adIs1240*[P*_eat-4_*::GFP]}, which was subsequently crossed with UA198 to produce UA360 {*sid-1*(*pk3321*); *baIn53*[P*_eat-4_*::*sid-1*, P*_dat-1_*::GFP]; *adIs1240*[P*_eat-4_*::GFP]; *baIn34*[P*_eat-4_*::Aβ,P*_myo-2_*::mCherry]}. The RNAi-sensitive UA198 was then crossed with each of the Aβ+ApoE strains to produce UA364 {*sid-1*(*pk3321*); *baIn53*[P*_eat-4_*::*sid-1*, P*_dat-1_*::GFP]; *adIs1240*[P*_eat-4_*::GFP]; *baIn50*[P*_eat-4_*::*APOE*ε2, P*_unc-54_*::tdTomato]; *baIn34*[P*_eat-4_*::Aβ,P*_myo-2_*::mCherry]}, UA365 {*sid-1*(*pk3321*); *baIn53*[P*_eat-4_*::*sid-1*, P*_dat-1_*::GFP]; *adIs1240*[P*_eat-4_*::GFP]; *baIn51*[P*_eat-4_*::*APOE*ε3, P*_unc-54_*::tdTomato]; *baIn34*[P*_eat-4_*::Aβ,P*_myo-2_*::mCherry]} and UA366 {*sid-1*(*pk3321*); *baIn53*[P*_eat-4_*::*sid-1*, P*_dat-1_*::GFP]; *adIs1240*[P*_eat-4_*::GFP]; *baIn52*[P*_eat-4_*::*APOE*ε4, P*_unc-54_*::tdTomato]; *baIn34*[P*_eat-4_*::Aβ,P*_myo-2_*::mCherry]}.

### Neurodegeneration analysis

Animals for analysis were synchronized with a 3-h egg lay using gravid hermaphrodites and incubated at 20°C, unless otherwise specified. To examine the neurons, hermaphrodites at indicated post-hatching time points were immobilized using 3 mM levamisole on glass cover slips and inverted onto 2% agarose pads on microscope slides. Each analysis was replicated at least three times with 30 animals per condition (30 animals×3 trials=90). *C. elegans* glutamatergic neurons were analyzed for neurodegeneration as previously described (Matlack et al., 2014; Tardiff et al., 2012, 2017; Treusch et al., 2011). Briefly, animals were scored for glutamatergic neurodegeneration at days 3 and 7 post-hatching, as reported in the Results and in figure legends. An animal was scored as normal if all five tail neurons were present and without malformities such as distention, apoptotic swelling, axon breaks, separation of the soma or loss of fluorescence.

### Mechanosensation assay

Assays were performed as previously described (Chalfie and Sulston, 1981; Chalfie et al., 1985). Briefly, animal populations were synchronized by a 3-h egg lay and progeny were incubated at 20°C until day 4 post-hatching. *C. elegans* sensitivity to soft touch was assayed by gently stroking hermaphrodite animals on the posterior and anterior with an eyelash hair glued to the end of a Pasteur pipette. Backward locomotion was induced by gently stroking the head of the animal with the eyelash followed by stroking the tail just below the anus. A positive result for soft touch sensitivity was recorded if the animal ceased backward locomotion or began moving forward. This process was repeated five times per animal, and the number of positive responses to posterior soft touch out of five was recorded. A total of 30 worms per strain were scored per biological replicate and percentage posterior touch response was calculated as the percentage average response within the population. The experiment was repeated at least three times (*n*=3×30=90) and data represent the average of all three biological replicates with s.e.m., as previously reported (Zhang et al., 2004).

### RT-qPCR

Quantitative PCR reactions were performed using IQ SYBR Green Supermix (Bio-Rad, Hercules, CA, USA) with the CFX96 Real-Time System (Bio-Rad) as described previously (Thompson et al., 2014). The following primer sequences were used: *APOE* Forward 5′-cctggacgaggtgaaggagca-3′, Reverse 5′-ctcgaaccagctcttgaggc-3′; *tba-1* Forward 5′-atctctgctgacaaggcttac-3′, Reverse 5′-gtacaagaggcaaacagccat-3′; *snb-1* Forward 5′-ccggataagaccatcttgacg-3′, Reverse 5′-gacgacttcatcaacctgagc-3′. Full-length gene sequences were obtained from WormBase and primers were evaluated for potential secondary structures of the amplicon by MFOLD software (http://unafold.rna.albany.edu/?q=mfold). MFOLD analysis was performed by adjusting the values to 50 mm Na^+^, 3 mm Mg^2+^ and 60°C annealing temperature.

At least 100 animals from each strain {UA356 [*adIs1240*(P*_eat-4_*::GFP+*lin-15*(+)); *baIn50*(P*_eat-4_*::*APOE*ε2, P*_unc-54_*::tdTomato)], UA357 [*adIs1240*(P*_eat-4_*::GFP+*lin-15*(+)); *baIn50*(P*_eat-4_*::*APOE*ε3, P*_unc-54_*::tdTomato)] and UA358 [*adIs1240*(P*_eat-4_*::GFP+*lin-15*(+)); *baIn50*(P*_eat-4_*::*APOE*ε4, P*_unc-54_*::tdTomato)]} were cultivated at 20°C, collected and RNA was harvested by Tri Reagent (Molecular Research Center, Cincinnati, OH, USA), according to the manufacturer’s guidelines. Following DNase treatment (Promega, Madison, WI, USA), cDNA strands were generated using the iScript cDNA synthesis kit (Bio-Rad). PCR efficiency was calculated from standard curves that were generated using serial dilutions of cDNA of all samples. Amplification was not detected in no template and no reverse transcriptase controls. The Cq quantification cycle values were recorded and consolidated by CFX Manager Software version 3.0 (Bio-Rad), then exported to Prism for one-way ANOVA. These data are represented as the mean of three biological replicates per targeted gene, each with three technical replicates and s.e.m. to represent the true mean of the populations. Reference genes *tba-1* and *snb-1* were used as internal controls. Relative mRNA expression levels were normalized using these reference control genes.

### RNAi

RNAi feeding clones were cultivated initially on LB solid medium containing tetracycline (5 μg/ml) and ampicillin (100 μg/ml) and then individual colonies were grown overnight in liquid LB medium containing 50 μg/ml carbenicillin. Isopropyl β-D-1-thiogalactopyranoside (IPTG) was spread on plates to a final concentration of 100 μM, seeded with RNAi feeding clones and allowed to dry. Induction of dsRNA occurred during a 14- to 18-h incubation at 20°C. Adult hermaphrodites were allowed to lay eggs for 3 h on RNAi feeding clones to produce a synchronized population. Glutamatergic neurons of synchronized progeny were analyzed at least 2 days after hatching, as described above.

### L1 starvation

Age-synchronized populations of each *C. elegans* strain were generated by bleaching. Briefly, 10 ml bleaching solution (1 ml 5N KOH, 2 ml bleach, 7 ml ddH_2_O) was used to isolate embryos. Embryos were then washed three times in 10 ml 1× M9 buffer to remove bleaching solution. Embryos were then transferred to standard NGM plates seeded with 200 μl OP50 bacteria or NGM plates containing no bacteria. After 24 h, animals were moved to NGM plates seeded with 200 μl OP50. Animals were incubated at 20°C for a total of 96 h and then 90 worms per strain were assayed for neurodegeneration.

### Peptone absence

Animals and media were prepared as previously described (Greer and Brunet, 2009). Briefly, age-synchronized populations of animals were obtained by allowing gravid adults to lay eggs for 3 h at 20°C on NGM plates containing either the standard quantity of peptone (2.5 g/l), as our control, or no peptone, and seeded with OP50 at a concentration of 5×10^12^ CFU/ml. Animals were maintained at 20°C and transferred as necessary until scoring for neurodegeneration.

### Dietary deprivation

Animals and media were prepared as previously described (Lee et al., 2006). Briefly, age-synchronized populations of animals were obtained by allowing gravid adults to lay eggs for 3 h at 20°C on seeded NGM plates. Animals were transferred to fresh seeded plates as necessary until day 2 of adulthood (day 5 post-hatching), at which point they were either transferred to seeded plates as they had been previously (*ad libitum* condition) or transferred to unseeded plates (dietary deprivation). Animals were maintained for neurodegeneration analysis at day 7 post-hatching.

### Survival assays

Survival assays were performed as previously described (Hsin and Kenyon, 1999). Briefly, strains were allowed to grow at 20°C in optimal growth conditions for at least two generations before the experiment began. Synchronized animal populations for survival analysis were generated by a 1-h egg lay using gravid hermaphrodites and incubated at 20°C. The L4 molt was defined as *t*=0, at which time animals were transferred to experimental plates. A total of 200 animals for each of nine strains were examined and all strains were assigned five initial plates with 40 worms each. Animals were then transferred to new plates every day, until the worms' reproductive stage had passed, after which point animals were then transferred every other day to ensure that appropriate amounts of food remained on the plate. Survival was assessed immediately after each transfer, as previously described (Hsin and Kenyon, 1999). To score for death, animals were examined for locomotive response to prodding with a platinum wire. Briefly, animals were touched five times on the head and the tail and assessed for reverse or forward locomotion in response. Animals were classified as dead if they ceased moving and failed to respond to this stimulation. A third category, censored, was utilized for animals that did not die of the natural aging process. Worms were classified as censored if they crawled off the plate, burrowed, or displayed vulval rupture or internal hatching, as previously described (Hsin and Kenyon, 1999). Seeded plates were stored at 20°C until completion. In GraphPad Prism software, the log-rank (Mantel–Cox) method was used to account for differences between survival curves. Specifically, all time points are assigned equal weights in statistical calculations whereby the entire pattern or path of the curve is being analyzed in testing for significance, not just the maximum value displayed (Hansen et al., 2008).

### Pharmacological treatments

Thapsigargin (Acros Organics) was dissolved in dimethyl sulfoxide (DMSO) and added to NGM plates to a final concentration of 3 μg/ml, as reported previously (Zwaal et al., 2001), with the modification that thapsigargin was added directly to the medium rather than supplemented on the surface.

### Experimental design and statistical analysis

Hermaphrodites were analyzed, which is standard in the *C. elegans* field, and all animals were incubated at 20°C, unless otherwise specified. In all cases, sample sizes (typically 30 animals per condition; for a total of 90 animals) were standardized within each experiment and examined in a uniform fashion. All experiments used at least three independent replicates per experiment per variable to generate a mean and s.d. In experiments using one independent variable across multiple tested effects (e.g. neuron cell death as a function of construct type), a one-way ANOVA series was used with a multiple-comparisons post hoc test (Tukey's). For grouped analyses, a two-way ANOVA series was used with Sidak's post hoc test. Survival was analyzed by the log-rank (Mantel–Cox) method, as previously described in the survival assay section. *P*<0.05 was the absolute minimum threshold for statistical significance. Statistics were performed using GraphPad Prism software.
